# Understanding the Therapeutic Action of Antipsychotics: From Molecular to Cellular Targets With Focus on the Islands of Calleja

**DOI:** 10.1093/ijnp/pyae018

**Published:** 2024-04-17

**Authors:** Merve Direktor, Peter Gass, Dragos Inta

**Affiliations:** RG Animal Models in Psychiatry, Department of Psychiatry and Psychotherapy, Central Institute of Mental Health, Medical Faculty Mannheim, Heidelberg University, Germany (Mrs Direktor and Dr Gass); RG Animal Models in Psychiatry, Department of Psychiatry and Psychotherapy, Central Institute of Mental Health, Medical Faculty Mannheim, Heidelberg University, Germany (Mrs Direktor and Dr Gass); Translational Psychiatry, Department of Community Health , and Food Research and Innovation Center (FRIC); University of Fribourg, Switzerland; Department of Biomedicine, University of Basel, Switzerland

**Keywords:** Schizophrenia, negative symptoms, adult neurogenesis, ventral striatum, dopamine

## Abstract

The understanding of the pathophysiology of schizophrenia as well as the mechanisms of action of antipsychotic drugs remains a challenge for psychiatry. The demonstration of the therapeutic efficacy of several new atypical drugs targeting multiple different receptors, apart from the classical dopamine D2 receptor as initially postulated unique antipsychotic target, complicated even more conceptualization efforts. Here we discuss results suggesting a main role of the islands of Calleja, still poorly studied GABAergic granule cell clusters in the ventral striatum, as cellular targets of several innovative atypical antipsychotics (clozapine, cariprazine, and xanomeline/emraclidine) effective in treating also negative symptoms of schizophrenia. We will emphasize the potential role of dopamine D3 and M4 muscarinic acetylcholine receptor expressed at the highest level by the islands of Calleja, as well as their involvement in schizophrenia-associated neurocircuitries. Finally, we will discuss the implications of new data showing ongoing adult neurogenesis of the islands of Calleja as a very promising antipsychotic target linking long-life neurodevelopment and dopaminergic dysfunction in the striatum.

Despite decades of research, the pathophysiology, that is, the molecular and cellular substrates of schizophrenia, are still incompletely understood, hampering the development of more effective therapies. Deciphering the mechanism of action of antipsychotics may provide hints about brain structures implicated in schizophrenia. This would be particularly important regarding negative symptoms and cognitive deficits, which are still difficult to treat.

Based on the observation that the first, so-called typical antipsychotics identified antagonize dopamine D2 receptors (D2Rs), research focused for a long time on D2Rs as the presumed sole molecular determinant of the antipsychotic action. However, D2Rs are widely distributed throughout the brain, making it difficult to associate them with a certain disease-related region. Moreover, metoclopramide, a classical antiemetic, although also antagonizing D2Rs, does not elicit antipsychotic effects. Finally, the numerous, partly more efficient “atypical” antipsychotics developed more recently bind much less to D2Rs but to a plethora of different other receptors. This evolution complicates even more the understanding of the mechanisms of the disease and the current therapeutic principles, questioning D2Rs as the unique molecular target in schizophrenia but without offering instead a comprehensive, unified framework.

Early studies analyzing c-Fos expression, an intermediate-early gene used to map stimulus-induced neural activation, indicated the nucleus accumbens shell (NAcSh) as “common cellular action site” of all antipsychotics (Ananth et al., 2001). Accordingly, metoclopramide induces c-Fos in the nucleus accumbens core but not in the NacSh (Deutch et al., 1996). However, the NAcSh may not be the “holy grail” substrate of the antipsychotic effect: it may be related to clinical improvement only of positive symptoms (an effect of typical and atypical antipsychotics) but not of negative symptoms of schizophrenia (an attribute of only some few atypical neuroleptics) (Ananth et al., 2001). Interestingly, several innovative atypical antipsychotics (clozapine, cariprazine, and xanomeline/emraclidine) that are effective in treating not only positive symptoms but also negative symptoms target preferentially another, yet poorly studied brain structure, the islands of Calleja (ICj). Here we briefly review the data on the structure, development, and function of the ICj and evidence linking the ICj and the 3 antipsychotics mentioned and discuss implications for schizophrenia.

The ICj are located in the ventral striatum and represent unique clusters of densely packed small GABAergic granule cells, generated mainly at “late,” early postnatal stages and migrating from the subventricular zone (SVZ), similar to other granule cell-like GABAergic interneurons in the cortex and striatum (De Marchis et al., 2004; Inta et al., 2008). Granule cells surround larger neurons in the hilus of the islands. The ICj comprise a single larger island, the insula magna (ICjM), adjacent to the NAcSh, and numerous small islands (ICjs) scattered more ventrally (Fallon et al., 1978). Interestingly, the ICj, although apparently separated into distinct cell clusters, represent in fact—as shown by a 3-dimensional reconstruction—a massive, single, continuous cellular ‘“syncitium”” (de Vente et al., 2001) interconnected by gap junctions (Halliwell and Horne, 1998). This results in a unitary fast response of all ICj and may imply a high functional impact. One important recent study demonstrated that optogenetic activation of dopamine D3 receptor (D3R) expressing neurons in the ventral striatal ICj robustly induces orofacial grooming in mice (Zhang et al., 2021), a result potentially relevant for the self-care/hygiene deficits accompanying negative symptoms of schizophrenia. Additionally, the same group established self-grooming as a means of promoting social attraction among mice via volatile cues (Zhang et al., 2022). Finally, these authors revealed for the first time a crucial role of ICj D3R granule cells in bidirectionally mediating depression-like behaviors (Zhang et al., 2023).

The ICj are densely interconnected bidirectionally with dopaminergic neurons of the substantia nigra (SN) and ventral tegmental area (VTA), representing an important modulator of dopamine levels in the striatum (Fallon et al., 1978). They are devoid of D2Rs but have the highest density of D3Rs in the brain (Bouthenet et al., 1991). D3Rs, although displaying a high degree of sequence homology with D2Rs, have a completely different expression pattern, restricted mainly to neurogenic sites, like the SVZ and ICj, in contrast to the widespread D2R expression throughout the brain (Diaz et al., 1997). Activation of D3R leads to enhancement of gap junctional coupling between ICj granule cells in vitro (Halliwell and Horne, 1998), indicating a main role in regulating their function.

Clozapine, considered the gold standard in the treatment of therapy-resistant schizophrenia, activates specifically these brain regions, but strikingly, the neuronal activation pattern differs considerably depending on treatment length. Whereas acute administration induces c-Fos in all ICj and shNAc, it persists only in the ICjs following chronic treatment (Hurley et al., 1996). This is important on one hand, because chronic antipsychotic treatment resembles more realistically the clinical situation than a single administration. On the other hand, the ICjs receive a topographically distinct dopaminergic input from the SN than the ICjM, which is linked especially to the VTA (Fallon et al., 1978). Therefore, the exclusive activation of the ICjs by chronic clozapine indicates the SN and nigrostriatal pathways as targets of clozapine, a result of relevance, considering their increasingly postulated role in schizophrenia (MacCutcheon et al., 2019). The molecular mechanisms underlying the clozapine-induced activation of the ICjM are still poorly studied. Interestingly, c-fos expression following clozapine administration is absent in mice lacking the D3R (Robertson et al., 2004). Moreover, clozapine increased D3R mRNA levels by 5-fold without affecting any other dopamine receptors (Buckland et al., 1993). The potential implication of other (e.g., serotonin) receptors targeted by clozapine in the activation of the ICj was not yet determined. One neuroimaging study reported that clozapine-induced neuroanatomical remodeling differentially in wild-type (WT) compared with D3R KO mice, increasing the volume of the prelimbic (PL) area of WT mice at trend level but decreasing D3KO PL area glial cell density (Guma et al., 2019). However, changes in the ICj were not assessed, possibly due also to the difficulties in quantifying morphological changes in the complex, 3-dimensional structure of the ICj.

The more recently developed cariprazine is a third-generation antipsychotic agent with a unique D3R-preffering mechanism of action, showing a potency for the D3R higher than that exhibited by dopamine itself, leading to full D3R occupancy at clinically relevant doses (Calabrese et al., 2020). Importantly, unlike older antipsychotics, cariprazine is also effective against negative symptoms of schizophrenia, which remains an important area of unmet treatment need (Németh et al., 2017, 2022). Moreover, the beneficial effect of cariprazine on negative symptoms was reported to be fast, starting from 1 to 2 weeks of treatment and useful for determination of early clinical predictors for efficacy (Ivanov et al., 2022). A new study using the innovative PharmacoSTORM super-resolution imaging that enables cell type– and compartment-specific nanoscale molecular measurements provides in vivo evidence that cariprazine binds almost exclusively to the granule cells of the ICj and that this binding is absent in D3R KO mice (Prokop et al., 2021). This result is remarkable, since binding of cariprazine to other D3R-expressing brain regions or onto dopaminergic terminals, where D3 autoreceptors were reported previously (Diaz et al., 2000), was much lower or even absent (Prokop et al., 2021). It indicates that the ICj is the main cellular target of cariprazine. The mechanisms underlying the therapeutic superiority of cariprazine compared with classical neuroleptics is unknown, but considering these results it most likely involves D3R/ICj.

Xanomeline is a highly promising selective M1- and M4 muscarinic acetylcholine receptor (mAChR) modulator and therefore the first nondopaminergic antipsychotic under development. Its antipsychotic effect was discovered serendipitously, as it was aimed initially in the 1990s to treat cognitive decline in patients with Alzheimer disease and found to improve psychotic symptoms in patients with dementia. Despite having nearly equal in vitro potency at the M1 and M4 mAChRs, xanomeline elicits its effects in vivo preferentially via M4 receptors (Thorn et al., 2019). Moreover, the highly selective M4 receptor positive allosteric modulator emraclidine represents a very promising new antipsychotic as well (Krystal et al., 2022). Interestingly, by far the most intense expression of M4 mAChR in the forebrain of both rats and monkeys is in the ICj, where it appears to be a selective marker for the hilus regions of the islands (Wirtshafter and Osborn, 2004). The neuronal elements in the ICj, which express M4 receptors, remain to be identified. Nevertheless, due to the predominant expression in the ICj, they may play a main role in the antipsychotic action of xanomeline and emraclidine. One aspect that needs to be mentioned here is that xanomeline is administered within the dual novel antipsychotic KarXT together with the muscarinic antagonist trospium chloride to reduce peripheral cholinergic effects of xanomeline (Brannan et al., 2021). This raises the possibility that trospium chloride may have a balancing effect in the brain and influence the antipsychotic effect. However, unlike other antimuscarinic drugs, trospium chloride is hydrophilic and does not cross the normal blood-brain barrier in significant amounts and, therefore, has minimal central anticholinergic activity (Staskin et al., 2010).

Altogether these data indicate that despite different molecular targets, these antipsychotics with improved therapeutic potency also against negative symptoms may act predominantly on the same cellular substrate, the ICj (Figure 1). We proposed more than a decade ago that the ICj may represent a key structure in the pathophysiology of schizophrenia, linking impairment of “late” postnatal neurogenesis with dopaminergic dysregulation in the striatum (Inta et al., 2011). Moreover, recent data show that the neurogenesis of ICj granule cells is not restricted to early postnatal stages but continues into adulthood and even during aging, as demonstrated in mice (Haruyama et al., 2019) (Figure 1). This raises the exciting possibility that these antipsychotics exert their therapeutic action by modulating neuronal renewal of the ICj and associated changes of striatal dopamine metabolism throughout life.

**Figure 1.**
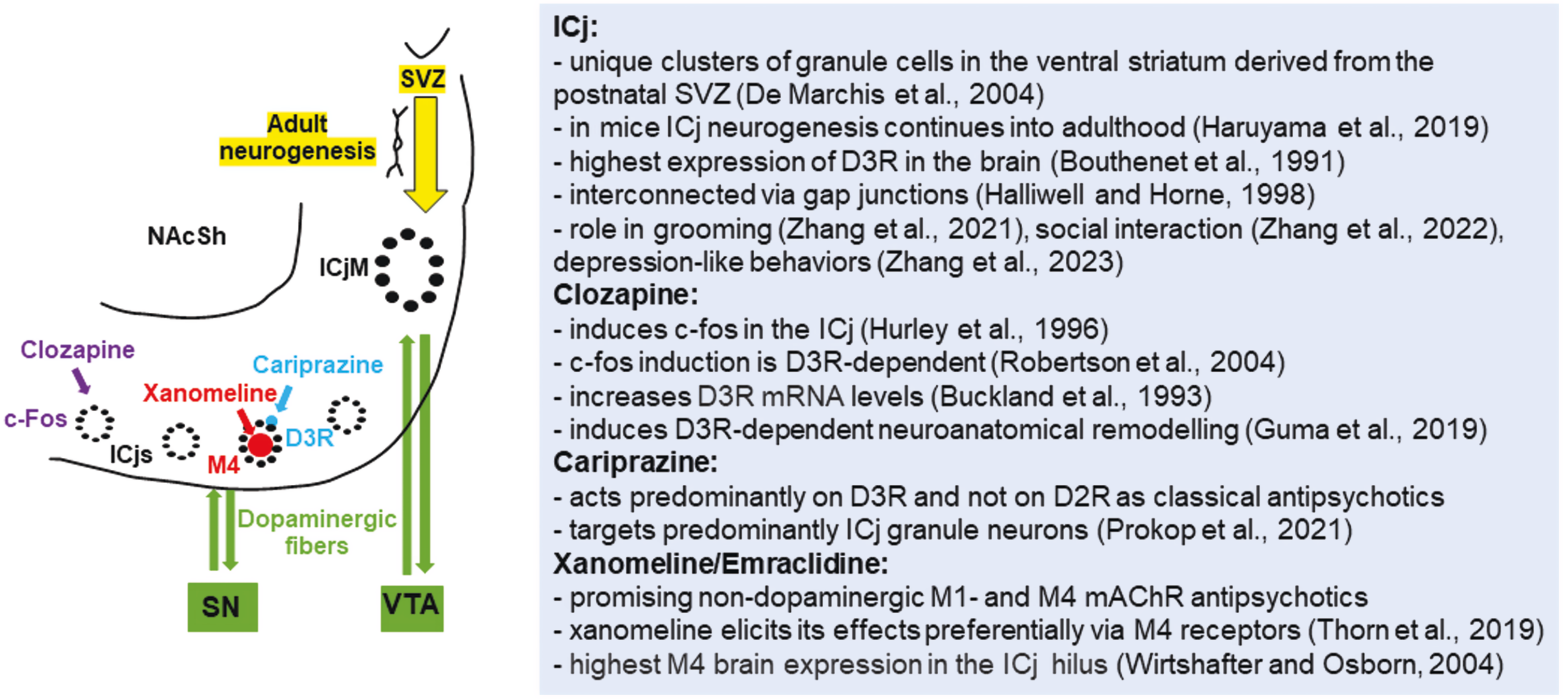
Schematic representation of the location, connectivity, and targeting by antipsychotics of the ICj and summary of the main results discussed. The topographically separated bidirectional dopaminergic projections from the SN to ICjs and VTA to ICjM are represented in green, the c-fos induction in the ICjs by chronic clozapine in purple, the binding of xanomeline to M4 muscarinic acetylcholine receptors on hilar neurons red, and that of cariprazine to D3R on granule cells in blue. The adult neurogenesis of the ICj and migration from the SVZ is depicted in yellow. ICjs, small islands of Calleja; ICjM, major island of Calleja, SN, substantia nigra; VTA, ventral tegmental area; NAcSH, nucleus accumbens shell.

Importantly, the ICj should not be considered as the only cellular structure involved in the antipsychotic effect. On the contrary, it is more likely part of a polysynaptic circuit including the ventral hippocampus, which was postulated to be involved in schizophrenia (Sonnenschein et al., 2021). This link is sustained by data showing that the classical neonatal ventral hippocampal lesion model of the disease strongly impairs only the expression of ICj D3R among all dopamine receptors (Flores et al., 1996). One interesting question is if other emerging new non-D2R binding antipsychotics also target the striato-hippocampal neurocircuitry comprising the ICj. In this respect, the trace amine-associated receptor 1 (TAAR1) agonist ulotaront that is effective in schizophrenia (Correll et al., 2021) was shown to modulate striatal and hippocampal glutamate function in a state-dependent manner (Yang et al., 2023). Another promising candidate, the voltage-gated sodium channel inhibitor evenamide, with efficacy in treatment-resistant schizophrenia, is a selective modulator of glutamate release (Singh et al., 2024); the brain regions implicated in its therapeutic action may be identified by subsequent studies.

In summary, the function of the different cellular compartments of the ICj (granule cells vs hilar neurons) as well as the role of the antipsychotic targets expressed by them (D3R, M4 mAChR) is still understudied. Future research focusing not only on molecular targets of antipsychotics but also on still insufficiently studied cellular substrates, like the ICj, and their role in schizophrenia-associated neural circuitry may provide new clues and potentially a more integrative pathophysiological view, allowing the future development of improved therapies.

## Data Availability

No new data were generated or analyzed in support of this research.
